# Competence expected from advanced-level paramedics by emergency medical services managers in Finland: a modified Delphi study

**DOI:** 10.1186/s13049-025-01384-5

**Published:** 2025-04-15

**Authors:** Antti Tanninen, Anne Kouvonen, Hilla Nordquist

**Affiliations:** 1 Department of Public Health, University of Helsinki, Helsinki, Finland; 2 Faculty of Social Sciences, University of Helsinki, Helsinki, Finland; 3 Centre for Public Health, Queen’s University Belfast, Belfast, Northern Ireland; 4 South-Eastern Finland University of Applied Sciences, Kotka, Finland; 5 LAB University of Applied Sciences, Lappeenranta, Finland

**Keywords:** Paramedics, Professional competence, Emergency medical services, Support, Emergency nursing, Leadership

## Abstract

**Background:**

Competence is a critical attribute for paramedics in emergency medical services (EMSs) because of the complex and diverse demands of the prehospital environment. This study aimed to identify and rank the key competencies expected of advanced-level paramedics as perceived by EMS managers.

**Methods:**

The modified Delphi study included three rounds conducted between October 2022 and June 2023. The panel consisted of 44 EMS managers, all working as superiors in EMS organizations across Finland.

**Results:**

In Round 1, 44 experts (100% response rate) evaluated 43 claims, with a consensus (≥ 75%) reached on five claims. The open-ended responses generated seven additional claims. In Round 2, 45 claims were reviewed; however, no consensus was reached. In Round 3, the top 15 claims from previous rounds were reevaluated (95% response rate), with assessment of patient conditions via the ABCDE protocol ranking highest. The experts also emphasized patient-centered care, safe environments, and systematic approaches in EMS.

**Conclusions:**

This study used the Delphi method to identify essential competencies for advanced-level paramedics, emphasizing patient assessment via the ABCDE protocol. This highlights the importance of core skills and nontechnical competencies such as supervision and well-being, stressing the need for continuous updates in paramedic training.

## Background

Competence is a key attribute for paramedics in emergency medical services (EMS) because of the complex and diverse demands of the prehospital setting [1]. Their working environment varies significantly, as they work primarily in private homes and public places [1]. The working hours of paramedics usually range from 12- to 24-hour shifts. They are on call during their shift, maintaining readiness to respond to given missions, and their work consists of multidimensional competencies, including cognitive, functional, and personal aspects [2]. Paramedics’ work also requires good skills in various procedures, such as sedation, intubation, and cricothyrotomy [3, 4]. In addition, EMS work often requires good team working skills, and in some countries such as Finland, which is the country setting of this study, advanced-level paramedics usually work in pairs in ambulances. Considering the high demands of the prehospital setting, competence plays a vital role in the EMS.

Paramedics’ wide scale of competencies is seen as the comprehensive ability to treat and diagnose different conditions outside the hospital setting. For example, von Vopelius-Feldt et al. [5] identified 389 different competencies in their study in England. The complexity of the paramedic profession is explained through the existence of multiple core competence areas and a wide variety of single competencies [6]. Furthermore, a Swedish study identified medical care, contextual aspects, and nursing as paramedic competence areas that EMS managers desire [7]. In another Swedish study, the main emphasis was on medical knowledge rather than other competencies in paramedic university-level training [8]. Bremer et al. [9] validated and translated the global rating scale of clinical competencies for paramedics, which consists of seven dimensions leading to overall clinical performance. Another validated scale was developed by Nilsson et al. [10] called the Ambulance Nurse Competence (ANC) scale, which results in the following eight factors: Nursing Care, Value-based Nursing Care, Medical Technical Care, Care Environments Community, Care Environments Serious Events, Leadership Management, Supervision and Professional Conduct, and Research and Development.

The present study was conducted in Finland, where health and social care regions have a legal obligation to organize emergency medical services (EMSs). EMSs must be planned and executed in collaboration with other healthcare services to ensure that services typically provided at patients’ homes form a cohesive regional and functional entity. Service standard decisions specifically define the content of the service and paramedics’ required level of training, the timeline in which the EMS must reach most of the population in the area, and other significant matters related to the EMS [11]. EMS personnel in Finland are highly educated. In 2021, there were approximately 3,900 paramedics in Finland, of which about 67% are advanced-level and 33% are basic level [12]. By law, an advanced-level paramedic must have a four-year bachelor’s degree in health care specializing in emergency care or a bachelor’s degree in nursing with at least 30 European credit transfer system credits (ECTS) of additional training focused on advanced-level emergency care [13].

For advanced paramedics, the need to have a wide variety of competences is evident. However, the order of importance of these competencies has not been sufficiently explored. Our study aimed to examine the desired competence of advanced-level paramedics from the Finnish EMS managers’ point of view.

## Methods

This study was carried out via the modified Delphi technique, which involves gathering the views of the subject expert panel [14]. In the Delphi method, the key idea is that group opinions are more valid than individual opinions are. This method has been widely used in previous international studies investigating paramedic competence and work [1, 6, 7, 15].

The inclusion criterion for the expert panel was that the expert was working in a supervisory position at the time of the study or had been working in a supervisory position within the past two years. The experts were advanced-level paramedics and worked in EMS in both operational and administrative supervisory roles. The recruitment was targeted to ensure that the expert panel covered Finland geographically as comprehensively as possible and that their expertise was well balanced in relation to regional differences. Recruitment started in September 2022 by contacting Finnish EMS supervisors and managers via the National EMS Field Supervisors’ Association and EMS Managers’ national network. The experts were provided information about the study and data protection. Informed consent for participation was confirmed by email prior to sending the first questionnaire. In this study, the panel consisted of 44 EMS managers, all working as superiors in EMS organizations across the country. Background information collected from the experts included age, education, and professional title. The participants were anonymous to each other. We used the Webropol survey & reporting© software in the Delphi rounds.

### Round 1

The claims for Delphi Round 1 were formulated on the basis of the ANC scale [10]. The questionnaire included 43 items related to the following eight competency areas: nursing care, value-based nursing care, medical technical care, the care environment community, care environment serious events, leadership management, supervision and professional conduct, and research and development [10]. The expert panel members were asked what level of competence advanced-level paramedics should have for each of the 43 claims. They were asked to answer using a scale of 1–7 (1 = very poor, 2 = poor, 3 = fair, 4 = intermediate, 5 = very good, 6 = excellent, 7 = exceptional). In addition, there was an open-ended question for every competency area to determine whether the experts recognized related competencies that were not mentioned in the original ANC scale. The Round 1 questionnaire was sent out in early October 2022, and the round ended in January 2023. Two reminder messages were sent during this time.

Answers from Round 1 were recategorized from the original 1–7 scale to a new 1–5 scale; poor = 2 and fair = 3 were merged as poor = 2, and very good = 5 and excellent = 6 were merged as very good = 4. In Round 1, consensus was set to be reached if 75% of the answers concurred in any of the final five categories. The results from the open-ended questions were analysed via inductive content analysis [16] and formulated as statements for the next round.

### Round 2

In Round 2, the experts were given a compilation of the Round 1 results. The compilation contained a table showing the division of the answers regarding the claims in Round 1 and the claims that had reached consensus, as highlighted. The experts were then sent a personal link via email to the Round 2 questionnaire. In Round 2, the expert panel members were asked their opinions on those claims that did not reach consensus in Round 1. Round 1 open-ended question answers were transformed into new claims and were included in the Round 2 questionnaire. The Round 2 questionnaire was answered via a 1–5 scale (1 = very poor, 2 = poor, 3 = intermediate, 4 = very good, 5 = exceptional) and consisted of a total of 45 claims. The mean values of the claims were calculated.

### Round 3

In Round 3, the experts were not given a compilation of Round 2 answers. The experts were sent a personal link to the Round 3 questionnaire via email. The questionnaire consisted of one ranking-type question and one open-ended question. For the ranking question, 15 of the previous rounds’ (including Round 1) claims that reached the highest mean value were selected. The expert panel members were asked to rank the five most essential competencies that paramedics should have out of these 15 claims. They were also asked to justify their selection with an open-ended question.

The analysis was conducted by coding the rankings as numerical values as follows: the claim that was ranked first was given a numerical value of 5, the claim that was ranked second was given a value of 4, and so on until the claim that was ranked fifth received a numerical value of 1. The given numerical values were then summed under every claim, resulting in a ranking from 1st to 5th. The principles of inductive content analysis were adopted to analyse the results of the open-ended questions [16].

## Results

### Round 1

Forty-three claims were introduced to the experts (*n* = 44), and the response rate was 100%. Consensus (minimum 75%) was achieved for the following five claims (Table 1): take responsibility for in-depth and continuous supervision of students (81.8% responded very good); identify patients with risk behavior and interact with optimal care levels (77.3% very good); assess the patient’s condition according to ABCDE (protocol for assessing a patient’s condition in a specific order), carry out investigations, decide and evaluate interventions (76.8% exceptional); initiate and take responsibility for supervision in nursing care (75% very good); and organize nursing care to promote the well-being of patients and their close relatives (75% very good). Open-ended responses (*n* = 20) varied from short single sentences to more than 160 words long and were categorized into seven new claims: recognize gaps in their competence; take care of their physical functioning; can utilize their working life and interpersonal skills in the workplace; maintain their well-being at work; can handle the ambulance reliably in traffic; can act as a situation leader in EMS missions; and understand the operating principles of the EMS system. In total, 45 claims (38 claims not reaching consensus and 7 claims from the open-ended question) were moved to Round 2.

### Round 2

The response rate in Round 2 was 90.9%. None of the 45 claims reached the 75% consensus (Table 1).

The mean values of Rounds 1 and 2 varied from 4.72 to 3.05. The highest mean value (4.72) was found for the claim “assesses the patient’s condition according to ABCDE, carries out investigations, decides and evaluates interventions.” The 15 claims with the highest mean values are presented in Round 3 (Table 1).

**Table 1:** Results of the Delphi rounds

Results of the Delphi rounds On what level of competence advanced level paramedics should have (Advanced level paramedic should be able to… )	Round 1					Round 2 mean	Round 2 SD	Round 3 included (mean/SD)	Round 3 ranking (summed values)
	Very poor	Poor	Intermediate	Very good	Exceptional				
Apply a systematic approach in nursing care of sick and/or injured patients*	0.0%	0.0%	4.6%	47.7%	47.7%	4,53	0,55	6	4th (46)
Identify symptoms and signs of illness, promote well-being and prevent care related suffering	0.0%	0.0%	6.8%	61.4%	31.8%	4,38	0,67	14	(26)
Adjust the pace of care to sick and/or injured patients	0.0%	2.3%	13.6%	59.1%	25.0%	3,88	0,79		
Make use of the patients’ experience and knowledge ensuring that nursing care and treatment are based on the patients’ dignity and rights	2.3%	9.3%	20.9%	65.2%	2.3%	3,65	0,66		
Document, evaluate, and report the assignment ensuring patient safety	0.0%	0.0%	2.3%	45.4%	52.3%	4,48	0,51	10	5th (44)
**Organize nursing care to promote the well-being of patients and their close relatives**	0.0%	0.0%	15.9%	**75.0%**	9.1%				
Identify infection and carry out infection prevention in nursing care	0.0%	0.0%	18.2%	65.9%	15.9%	3,90	0,67		
Adjust information and education to sick and/or injured patients and their related persons	0.0%	4.6%	13.6%	72.7%	9.1%	3,95	0,64		
Promote patients’ and families’ involvement in health promotion and self-care	0.0%	18.2%	36.3%	43.2%	2.3%	3,30	0,72		
Lead nursing care of deceased patients and their closely related in cooperation with other professions	2.3%	22.8%	31.8%	38.6%	4.5%	3,35	0,83		
**Identify patients with risk behaviour and interact with optimal care levels**	0.0%	4.5%	11.4%	**77.3%**	6.8%				
Identify patients and related relatives suspected of being subjected to abuse or violence and report according to legislation	0.0%	0.0%	9.1%	72.7%	18.2%	4,08	0,66		
Identify ethical issues in relation to resource shortage and organize care	0.0%	15.9%	29.6%	50.0%	4.5%	3,30	0,61		
**Assess the patient’s condition according to ABCDE**, **carry out investigations**,** decide and evaluate interventions**	0.0%	2.3%	0.0%	20.9%	**76.8%**			1.(4.72/0.58)	1st (150)
Interpret values and vital parameters as a basis for decisions for health care interventions	0.0%	2.3%	0.0%	31.8%	65.9%	4,60	0,55	3	2nd (81)
Independently decide, administer, and evaluate pharmacological treatment based on local guidelines	0.0%	4.6%	4.5%	61.4%	29.5%	4,13	0,61		
Make use of medical technical equipment	0.0%	2.3%	4.5%	40.9%	52.3%	4,30	0,56		
Carry out and evaluate care and treatment based on care pace during transport	0.0%	0.0%	0.0%	45.5%	54.5%	4,53	0,51	7	(20)
Apply a professional approach with respect to the patient’s home environment	0.0%	2.3%	11.4%	68.1%	18.2%	4,00	0,56		
Apply ethical approach in nursing environment at the injury scene and in the public environment	0.0%	0.0%	13.6%	56.9%	29.5%	3,95	0,60		
Interact and communicate with representatives of the community and caregivers to ensure good and safe care	0.0%	2.3%	6.8%	63.6%	27.3%	4,18	0,59		
Move and transport sick and/or injured patients in a traffic and in a patient safe manner	0.0%	2.3%	0.0%	36.3%	61.4%	4,60	0,55	4	(27)
Make use of and apply information and communication technology	0.0%	2.3%	4.5%	56.8%	36.4%	4,30	0,56		
Identify risk environments and create safe care space	0.0%	0.0%	0.0%	47.7%	52.3%	4,48	0,55	11	(19)
Establish preparedness for threats and violent situations	0.0%	0.0%	2.3%	43.2%	54.5%	4,45	0,55	12	(5)
Carry out triage of patients in serious events	0.0%	2.3%	2.3%	38.6%	56.8%	4,49	0,60	9	(16)
Systematically plan and address ill health in connection with CBRNE injuries	0.0%	9.1%	29.5%	50.0%	11.4%	3,55	0,71		
Take responsibility for medical leadership, prioritization of care at the injury scene	0.0%	15.9%	18.2%	50.0%	15.9%	3,75	0,74		
Carry out triage to the relevant level of care in collaboration senior leaders	2.3%	7.0%	11.6%	62.8%	16.3%	3,83	0,71		
Collaborate with rescue service and police in serious event	0.0%	2.3%	4.5%	50.0%	43.2%	4,38	0,67	15	(24)
Initiate exchanges of experience between colleagues as well as lead reflective communication with colleagues	0.0%	4.6%	20.4%	54.6%	20.4%	3,90	0,71		
Respect and make use of associates’ knowledge and experience from care and rescue work toward common goal	0.0%	0.0%	7.0%	60.5%	32.5%	4,23	0,58		
Contribute to cost-effectiveness and optimal resource utilization in care	0.0%	18.3%	22.7%	45.4%	13.6%	3,43	0,81		
**Initiate and take responsibility for supervision in nursing care**	0.0%	6.8%	11.4%	**75.0%**	6.8%				
Apply an attitude that promotes the profession’s reputation and public confidence	0.0%	0.0%	2.3%	52.3%	45.4%	4,53	0,55	8	(25)
Understand the importance and consequences of following or deviating from treatment guidelines	0.0%	2.3%	0.0%	31.8%	65.9%	4,69	0,47	2	3rd (68)
**Take responsibility for in-depth and continuous supervision of students**	0.0%	0.0%	4.6%	**81.8%**	13.6%				
Identify knowledge gaps in ambulance care and contribute to clinical- and patient-related research	0.0%	15.9%	31.8%	50.0%	2.3%	3,33	0,62		
Implement evidence-based care	0.0%	4.6%	11.3%	65.9%	18.2%	4,08	0,73		
Take responsibility for developing information and communication support for the management of nursing data	2.3%	20.4%	43.2%	34.1%	0.0%	3,05	0,78		
Participate in the development of new technology and equipment	0.0%	27.2%	34.1%	36.4%	2.3%	3,08	0,66		
Identify shortcomings in relation to safety and contribute to safe and comfortable transportation	0.0%	2.3%	6.8%	54.5%	36.4%	4,30	0,65		
Share knowledge of the profession’s responsibilities to other professions, the general public and to patients	0.0%	11.4%	27.3%	54.5%	6.8%	3,53	0,78		
New statements formulated based on experts responses that were included in the second round:									
Recognizes gaps in their own competence						4,35	0,58		
Takes care of their own physical functioning						4,18	0,59		
Can utilize their working life and interpersonal skills at their workplace						4,18	0,50		
Maintains their own well-being at work						4,43	0,64	13	(17)
Can handle the ambulance reliably in traffic						4,55	0,50	5	(2)
Can act as a situation leader in EMS missions						4,00	0,64		
Understands the operating principles of the Emergency medical service system						4,23	0,70		

### Round 3

Fifteen claims with the highest mean value overall were reintroduced to the experts in Round 3. The response rate was 95%. The experts ranked the five most important claims as follows: (1) Assess the patient’s condition according to ABCDE, carry out investigations, decide on and evaluate interventions (27 experts ranked this claim as the most crucial); (2) Interpreting values and vital parameters as a basis for decisions for health care interventions; (3) Understand the importance and consequences of following or deviating from treatment guidelines; (4) A systematic approach in the nursing care of sick or injured patients; (5) Documents, evaluations, and reports of the assignment ensure patient safety. The summed values of the claims are reported in Table 2.

**Table 2:** Competence ranked by importance

Ranking the competencies by importance		
On what level of competence advanced level paramedics should have (Advanced level paramedic should be able to… )	Ranking	Summed values
Assess the patient’s condition according to ABCDE, carry out investigations, decide and evaluate interventions	1.	150
Interpret values and vital parameters as a basis for decisions for health care interventions	2.	81
Understand the importance and consequences of following or deviating from treatment guidelines	3.	68
Apply a systematic approach in nursing care of sick and/or injured patients*	4.	46
Document, evaluate, and report the assignment ensuring patient safety	5.	44
Move and transport sick and/or injured patients in a traffic and in a patient safe manner	6.	27
Identify symptoms and signs of illness, promote well-being and prevent care related suffering	7.	26
Apply an attitude that promotes the profession’s reputation and public confidence	8.	25
Collaborate with rescue service and police in serious event	9.	24
Carry out and evaluate care and treatment based on care pace during transport	10.	20
Identify risk environments and create safe care space	11.	19
Maintains their own well-being at work	12.	17
Carry out triage of patients in serious events	13.	16
Establish preparedness for threats and violent situations	14.	5
Can handle the ambulance reliably in traffic	15.	2

Most experts (79%) responded to the open-ended question in Round 3, clarifying the reason they ranked the five most important claims as they did. Seven justification categories were formed: patient-centeredness, safe care environment, understanding the whole EMS, systematic approach in patient examination, competence in basic EMS tasks, multiauthority cooperation skills, and occupational well-being. Below are a few examples of expert justifications:

*“I believe that the most important thing is mastering the basics and remembering that we are here for the patients.”* (Expert 5).

*“These are the core duties of a paramedic: to systematically assess the need for treatment and implement treatment on the basis of the findings.”* (Expert 14).

*“The most important thing in EMS is identifying the patient’s health problem and implementing appropriate treatment.”* (Expert 22).

## Discussion

We aimed to examine the desired competencies of advanced-level paramedics from the perspective of Finnish EMS managers via the Delphi method. The ANC scale developed by Nilsson et al. [10] was used as the framework in this study, as it broadly reflects the competence characteristics of Nordic prehospital care. The scale includes the following factors: Nursing Care, Value-based Nursing Care, Medical Technical Care, Care Environment Communities, Care Environment Serious Events, Leadership Management, Supervision and Professional Conduct, and Research and Development.

We found that consensus was reached on only five of the claims. Assessing the patient’s condition according to the ABCDE protocol, carrying out investigations, and deciding and evaluating interventions was the only claim reaching consensus at an exceptional level and was also ranked as the most critical. This result can be explained by the high value placed on basic competence within the paramedic field in general. Additionally, the responses to the open-ended questions strongly emphasized basic competencies, including patient examination and treatment, identification of critical injuries and symptoms, and recognition of emergencies.

The work of paramedics has expanded to encompass a more extensive range of services, necessitating skills that are effective in assessing and providing the required care. The aging patient population has also changed the nature of EMS [17]. Moreover, the number of EMS missions has notably increased [17–20]. Additionally, the cost-effectiveness of home-delivered services is likely to increase their utilization [21, 22], and it would be valuable to investigate how this will impact the work of paramedics in the future. In alignment with these changes, our results highlight the importance of interpreting values and vital parameters as a basis for making decisions regarding healthcare interventions.

Moreover, according to the answers to the open-ended questions, the most critical competencies are strongly related to the core skills required of paramedics. This finding is supported by Holmberg et al.‘s study [7], which identified “knowledge to assess the patient’s situation from a holistic perspective,” “medical knowledge to assess and care for different diseases,” and “knowledge to care for critically ill patients” as the top three essential competencies. This finding suggests that the skills required for the care of acutely ill patients continue to be highly valued even though paramedics’ competence set is broadening.

Our results indicated that understanding the consequences of following treatment guidelines or deviating from them was considered the third most important competence by EMS managers. This finding is supported by a systematic review [23], which revealed that adherence to treatment guidelines in out-of-hospital emergency care was variable. This variability was influenced by the evidence base of the guidelines, the quality of the recommendations, and their applicability. Focusing on these aspects would enhance the understanding of the importance of following treatment protocols. The EMS managers identified the ability to apply a systematic approach in the nursing care of ill or injured patients as the fourth most important competence for paramedics. Additionally, our results indicated that the fifth most important competence was documentation, evaluation, and reporting of assignments to ensure patient safety. Hagiwara et al. [24] reported that deviations from standard care practices or inadequate documentation were associated with an increased risk of adverse events in the prehospital setting, underscoring the critical importance of a systematic approach and precise documentation in EMSs.

The managers also identified several additional competencies not included in the ANC framework. These newly identified competencies included nontechnical skills such as taking care of one’s well-being at work and recognizing gaps in one’s skills. A previous English study [25] reported that health-related lifestyles impact paramedics’ well-being at work, supporting our observation of the importance of their ability to maintain their own well-being. Moreover, good workplace relationships are related to well-being at work, as identified in earlier research on paramedics [26]. According to a previous Irish study [27], the implementation of continuous learning models is considered necessary by paramedics. This finding supports our results related to recognizing personal deficiencies. Future research should explore how effectively paramedics can identify gaps in their competencies and their ability to respond to these observations.

In addition, the managers identified technical competencies, such as those related to handling and driving an ambulance. This finding is supported by a systematic review in which ambulance driving was identified as one of the fundamental components of emergency care and is broadly linked to paramedics’ skills across various categories, such as safety, risk assessment, and stress management [28].

Our results revealed that identifying patients with risky behavior, as well as interactions with optimal care levels, was desirable to achieve a very good level of risk. This competence has only been relatively recently described in the literature [10]. However, Wihlborg et al. [6] depicted similar competencies, such as considering the patient’s current life situation as a whole and correctly assessing threatening situations. Moreover, organizing nursing care to enhance the well-being of patients and their families achieved consensus. This could imply a future need for paramedic training to focus more on the comprehensive assessment of the patient’s treatment needs and mastery of a wider range of interventions.

When comparing the European Paramedic Curriculum (EPaCur) Framework with the findings of our study, several alignments can be identified. The aim of this framework is to establish comparable competency standards for paramedics on a European scale. The competencies ranked highest in our study are prominently featured in the EPaCur framework both at the knowledge and the skills levels. Systematic patient assessment and the interpretation of measurement results leading to decision-making were the two most highly ranked findings in our study, and they align closely with the competencies outlined in the framework. Additionally, the importance of reporting and documentation emerged as a key finding in our study. This is also addressed in the EPaCur framework, which emphasizes that paramedics should have a strong proficiency in these areas. Furthermore, there is a clear alignment in competencies related to patient safety and multiauthority collaboration, further reinforcing the relevance of these aspects both in our study findings and the EPaCur framework [29]. 

Paramedic training may be at a turning point in response to changing competency requirements and the evolving needs of fieldwork in EMSs. In Round 1, a consensus was reached on the claim that taking responsibility for the in-depth and continuous supervision of students is an essential competence. Similarly, Wihlborg et al. [6] reported that pedagogical aspects, such as supervising students, are fundamental competencies for paramedics. In parallel, the EPaCur framework defines as a general competence the ability to guide and supervise relevant personnel undergoing the learning process [29]. Given that clinical placements are an extensive part of training, the role of mentorship and supervision is likely to become even more requisite in the future. Areas for further research could include examining the implementation of workplace training in alignment with the desired competencies, with a focus on the development of pedagogical competence to enhance training effectiveness. Comparing the desired competencies with ongoing paramedic competence maintenance training could help clarify their relative importance. In addition, investigating practical strategies for supervisors to effectively implement these competencies would be valuable. Understanding paramedics’ perspectives on the most critical competencies in the EMS field could also offer important insights into their real-world applicability.

### Methodological considerations

One of the strengths of the Delphi technique is its ability to facilitate consensus among a group of experts while avoiding the influence of any single dominant panel member [30]. To truly enrich the panel and minimize bias in the Delphi process, it is essential to include experts with diverse backgrounds, ages, and genders. This approach introduces multiple perspectives, which can offer valuable insights and mitigate the risk of bias, as emphasized by Keeney et al. [31]. While there is no definitive sample size recommended for Delphi studies, panels of 10 to 50 participants are often suggested, depending on the study’s purpose [14]. Even though the 44 experts in this study varied in terms of background, gender, age, and geographic location, they were all Finnish EMS managers with paramedic experience. They can thus be considered a homogeneous group.

Boulkedid et al. [30] highlighted the importance of individual feedback, both quantitative and qualitative, in enhancing the Delphi process. In this study, feedback which summarized the results was provided in tabular form between rounds 1 and 2. Additionally, the participants received reminder messages to encourage responses and improve response rates. The response rate remained high across all rounds (100%, 90.9%, 95%) in this study. Owing to respect for the experts’ privacy, the reason for their withdrawal was not pursued.

The Delphi literature does not provide clear guidance on the appropriate level of consensus. A predetermined level of 75% was selected because it has been recommended by and commonly used in previous studies [14, 15, 31]. In this study, no further consensus was found for the claims in the round 2. The Round 3 approach, which used ranking and reasoning, resulted in identifying the five most important competencies expected from advanced-level paramedics from the viewpoint of EMS managers, our experts. It should be noted that the experts emphasized skills to some extent in their views, and additionally, the ANC scale is leaning towards skills, which can be considered as a limitation. Nevertheless, our study included 44 experts from various parts of Finland, a number and geographical coverage that provided a robust and comprehensive range of information. Still, the differences in EMS systems across different countries may limit the generalizability of the findings.

## Conclusions

We utilized the Delphi method to assess the competencies deemed indispensable for advanced-level paramedics from the perspective of EMS managers, using the ANC scale as a framework for this study. Consensus was achieved on five competencies, with assessment of the patient’s condition according to the ABCDE protocol being the highest. The findings emphasize the importance of fundamental paramedic skills, particularly in patient assessment and intervention. Additional competencies (such as supervision of students and nontechnical skills such as maintaining well-being) were also highlighted as crucial, suggesting the need for ongoing adaptation in paramedic training to meet the evolving demands of this field. Comparing these competencies with ongoing paramedic competence maintenance training could clarify their importance, while exploring strategies for effective implementation and paramedics’ perspectives would provide valuable real-world insights.

## Data Availability

The datasets used and/or analysed during the current study are available from the corresponding author upon reasonable request.
